# How Funding Policy Maintains Structural Inequity Within Indigenous Community-Based Organizations

**DOI:** 10.1377/hlthaff.2023.00483

**Published:** 2023-10

**Authors:** Arielle R. Deutsch, Leah Frerichs, Zeynep Hasgul, Foster Murphey, Addie K. Coleman, Annie Y. Bachand, Arlana Bettelyoun, Paul Forney, Gene Tyon, Mohammad S. Jalali

**Affiliations:** Avera Research Institute, Sioux Falls, South Dakota; University of South Dakota, Vermillion, South Dakota.; University of North Carolina at Chapel Hill, Chapel Hill, North Carolina.; Massachusetts General Hospital and Harvard University, Boston, Massachusetts.; Avera Research Institute.; University of North Carolina at Chapel Hill.; Urban Roots Ancient Wisdom, Rapid City, South Dakota.; Oglala Lakota Children’s Justice Center, Pine Ridge, South Dakota.; Avera Research Institute.; Oaye Luta Okolakiciye, Rapid City, South Dakota.; Massachusetts General Hospital, Harvard University, and Massachusetts Institute of Technology, Boston, Massachusetts.

## Abstract

Despite efforts to increase investment in Indigenous health and well-being in the United States, disparities remain. The way in which health-promoting organizations are funded is one key mechanism driving the systemic, long-term health disparities experienced by Indigenous people in the US. Using Indigenous-led community-based organizations (ICBOs) that provide psychosocial care as a case study, we highlight multiple ways in which policies that regulate the external funding that ICBOs depend on must change to promote equity and allow the organizations to flourish and address unmet psychosocial needs for Indigenous community members. We use a system dynamics approach to discuss how “capability traps” arise from a misfit between external funding regulations and organizations’ needs for sustainability and effective care provision. We provide suggestions for reforming funding policies that focus on improving ICBO sustainability.

Health funding and funding policies are key mechanisms of the systemic inequity driving the long-term psychosocial health disparities experienced by American Indian and Alaska Native (referred to here as “Indigenous”) communities.^1,2^ This has led to consistent calls for greater investment in Indigenous health^3^ and subsequent increases in research, infrastructure, and programmatic funding.^4,5^ For instance, in 2023 the Biden administration allocated almost $8 billion in funding for the Indian Health Service (IHS) and urban Indian health, the federal treaty–obligated health care providers for Indigenous people in the US. This budget included advance appropriations (in which funds are made to become available in the years after the appropriations act passes) for the first time, allocating $5 billion in funding beyond 2023.^6^ However, Indigenous populations continue to disproportionately experience greater challenges to their psychosocial well-being as a result of a legacy of historical trauma that is maintained by current systemic inequity and racism.^7^

Prior research on the effect of funding on these disparities has focused on IHS-provided care.^8,9^ There is less research on funding for the considerable amount of non-IHS psychosocial care provided for Indigenous people, including through community-based organizations (CBOs).^10^ Psychosocial care–providing CBOs are funded primarily through external service-focused public or private grants and subcontracted services^11^ and are an attractive strategy to improve health equity among many systemically marginalized populations,^12^ particularly given barriers that patients and providers face with IHS-provided care.^13^ Indigenous-led CBOs (ICBOs) can be better equipped to provide culturally grounded health resources that are demonstrably more effective for Indigenous communities compared with standard evidence-based practices.^14^ ICBOs also are able to build stronger authentic relationships with community members compared to larger public, nonprofit, or private institutions with ongoing legacies of racial mistreatment.^7^

The nonprofit sector has long discussed the negative consequences of the policies and practices that funding organizations use for grant making and grant regulation. However, little attention has been paid to the barriers that ICBOs face for maintaining funding, capacity, and infrastructure, which are rooted in structural racism and less likely to affect more established, White-led CBOs.^15^ Similar to challenges faced by CBOs led by other minoritized groups, ICBOs are highly dependent on external grants (directly or through subcontracts), given their low alternative funding streams (such as insurance reimbursement). Funds from reimbursed care are low: Indigenous people have high uninsurance rates,^8^ and Indigenous traditional healing approaches are not reimbursable through public insurance options in many states.^14,16^ The diverse needs and substantial trauma in many Indigenous communities pose additional barriers for ICBOs’ efforts to provide psychosocial care^15^ and retain operational health. Furthermore, finding appropriate staff members who are experienced in both local cultural practices and Western psychosocial care may also be challenging. ICBOs also must contend with unique jurisdictional and political complexities of health care (including psychosocial) provision that further burden operations.^17^ That is, the institutions that are responsible for managing and providing care (and therefore that have political and financial power) can differ between and within local areas, organizations, and Tribal affiliations. Although these issues challenge ICBOs’ sustainability,^18^ there is little discussion of how the policies regulating the external funding that ICBOs depend on contribute to this disparity.

The current article highlights how funders’ policies for grant making and regulation must change to equitably allow ICBOs to flourish and address unmet psychosocial needs for Indigenous communities. Prior research has criticized how external funding policies and practices based on what works for larger and well-funded organizations are incompatible with the needs of small, social justice–oriented CBOs.^19^ We argue that this incompatibility also contributes to maintaining structural racism and the overall oppression of Indigenous health.^20^ Specifically, our study used a system dynamics approach^21^ to explain how the misfit between external funding provision to ICBOs and their funding needs for efficacy and sustainability results in capability traps^22^ that inhibit ICBOs’ ability to thrive. Capability traps occur when short-term solutions, often in the form of “quick fixes,” are applied to problems at the expense of long-term improvement. Over time, these short-term solutions can erode the capability required for long-term sustainability, making it increasingly difficult to achieve desired goals or outcomes^23^ (in this case, sufficient funding to maintain service provision). Conversely, such funding policies help the larger, White-led organizations that dominate psychosocial care provision (including public and private health organizations, universities, and social welfare and criminal justice institutions) maintain their higher capacity, infrastructure, and sociopolitical capital. Finally, we suggest broad ways in which funding policy can change to improve how ICBOs can collaboratively mobilize to enhance community well-being.

## Study Data And Methods

The purpose of this article is to clarify how policies to fund psychosocial health care provided by ICBOs are a critical area of broader systemic inequity that must be addressed in order for programmatic efforts (the most common approach to addressing Indigenous psychosocial well-being) to be successful and sustainable. The findings in this article are further grounded in an ongoing community-based system dynamics project^24^ that focuses on improving strategies to address health inequity surrounding alcohol- and substance-exposed pregnancy and the synergistic interplay between alcohol and substance use, intimate partner violence, and unintended pregnancy^25,26^ within Indigenous communities. The goals of this project are to better understand the “real world” complex system that drives these issues by combining the different ways in which this system is perceived by community members with diverse relevant personal or professional experiences, and to identify highly effective and equitable strategies that can reduce both rates of and disparities in these issues.

### STUDY POPULATION

Our project participants include 148 members of a small metropolitan community in the Northern Great Plains region. The sample represents diverse personal or professional experiences within Indigenous health, partner violence, alcohol and substance use, and perinatal health. Most participants are female, and approximately 50 percent are Indigenous. Online appendix 1 provides detailed information on the sample, data collection, and methodology.^27^

### METHODS

Our project uses a system dynamics methodology,^21^ which is a modeling and simulation approach. Modelers elucidate how inter-actions between variables within a complex system drive patterns of behavior within the system through formal diagramming.^28^ Community-based system dynamics involves a participatory model-building process that is well suited to evaluating complex systems in contexts of structural inequity and racism.^29^ In our participatory model-building exercises, community member participants, who represented diverse areas of knowledge and experience,^30^ would individually or collaboratively build visual diagrams of the complex systems underlying the issues of interest (see appendix 1 for description of model-building session activities).^27^ The purpose of these exercises is to reflect the “real world” system that explains variables or issues of interest through the collective diverse perspectives of those that experience this system. Models are iteratively refined through multiple sessions with the same or new participants to ensure external validity. Causal loop diagrams illustrate system variable relationships and feedback loops.

The causal loop diagrams we discuss in this article were based on our participatory community stakeholder–developed models and modeling session notes. We focused on the variables and loops pertaining to funding of Indigenous psychosocial care from the broader systems models developed as part of the broader project. These funding-specific models were further refined through additional iterative discussion with collaborating Indigenous community partners (coauthors). All community coauthors had experience and expertise leading psychosocial care-providing ICBOs and navigating public, private, and Tribal care provision entities. Finally, we contextualized our results through examining prior research on CBO processes and psychosocial care for Indigenous communities. This study was approved by the Institutional Review Board of Avera Research Institute.

### LIMITATIONS

This study had several limitations. First, our models might not generalize to other communities with different regional policies (such as reimbursement for traditional Indigenous healing),^16^ community sizes, and resource accessibility. Second, findings might not generalize to ICBOs that do not focus on health and psychosocial care. Third, because this was a community-level overview of funding policy, we aggregated some more granular-level variables for clarity. Finally, variables that may intersect but are not the focal point of the current findings (such as person-level access to resources) also were excluded.

## Study Results

Modeling and iterative discussions with community members highlighted six relevant policies (exhibit 1) that public and private external funding institutions use to allocate and regulate funds. They are regulations on direct service expenditures; indirect cost rates; funder requirements for grant compliance; single-issue, direct-service focus; prioritization of Western evidence-based practices; and service provision as a success metric. These policies were common to diverse types of funding, from public to private and local to federal. Here we provide an overview of how these policies affect ICBOs at the organizational and community levels. Models are broken down by key feedback loops and are presented in appendix exhibits 2a-2d.^27^ Feedback loops are presented through overarching themes that contextualize how funding policies inhibit ICBOs’ sustainability and efficacy. These themes include within-ICBO funding cycles; evidence-based practice–related challenges; within-community, between-CBO competition; and within-community, between-CBO network collaboration. A full model detailing how all of the loops are connected is in appendix exhibit 3.^27^

Feedback loops visually represent a cycle of causal interrelationships between a set of variables, in which a change in the behavior of one variable in the loop facilitates a series of effects that returns to an effect on the initial variable. Relationships between variables have either positive (variables change in the same direction) or negative (variables change in opposite directions) valences. The valence of all relationships between variables creates the feedback loop polarity. In reinforcing feedback loops, an initial change in one variable in the loop prompts causal responses through the loop that result in eventually amplifying the direction of the change in the initial variable. In balancing feedback loops, an initial change in one variable in the loop prompts causal responses through the loop that counteract the direction of the initial change.

### WITHIN-ICBO FUNDING CYCLES

We identified four key feedback loops that drive funding cycles within ICBOs (appendix exhibit 2a).^27^ These loops emphasize how the capability trap that arises through the struggle for ICBOs to make up gaps in operational capacity through seeking grant funds results in ICBOs securing only enough funding to maintain operations but not close the gap completely. In the realm of grant funding, funders stipulate both acceptable direct costs and indirect cost rates (see exhibit 1 for operational definitions). ICBOs often start with low levels of operational capacity and infrastructure needed to be effective. Even after obtaining funds, ICBOs also typically have low negotiated indirect cost rates, therefore receiving insufficient indirect funds to close this gap (appendix exhibit 2a, loops A and B).^27^ The enduring gap between existing and needed capacity has long-term ramifications for sustainability. Costs required to provide care in high-trauma, low-resource communities (for instance, trust-building engagement with potential clients and community members) exacerbate this gap, as they are not always allowable direct service expenditures. The cost of providing these types of services requires ICBOs to use more of their indirect funding, further limiting funds that can be spent on building capacity and infrastructure. This gap is again reinforced as lower capacity reduces both the number and the size of grants that ICBOs can obtain, creating a cycle of constrained capacity building.

Grant applications are high-burden efforts that strain ICBOs’ existing infrastructure. ICBOs can often lack requisite knowledge and requirements (such as identifying funding opportunities or obtaining federal grant application accounts), as grant-writing information and resources are embedded in the networks of White-led institutions. ICBOs therefore require more (small-size) grants to maintain sustainability, further straining operations (appendix exhibit 2a, loop C).^27^ Managing grant funding then strains infrastructure, as grant management for multiple small grants often requires more effort and infrastructure than what indirect funding provides. Taken together, this cycle results in ICBOs obtaining “just enough” funding for survival, with little opportunity for growth (appendix exhibit 2a, loops D and E).^27^

### EVIDENCE-BASED PRACTICE–RELATED CHALLENGES

We identified three loops highlighting how funders’ prioritization of Western evidence-based practices also reduces capacity and infrastructure and the size of the grants that ICBOs can obtain (appendix exhibit 2b).^27^ ICBOs emphasize serving their own populations and using culturally grounded approaches to care. Many aspects of Indigenous traditional psychosocial care are not considered direct expenses within strict parameters of direct costs, as defined by Western evidence-based practices provided by large-capacity White-led organizations (appendix exhibit 2b, loop A).^27^ Therefore, ICBOs often partner with (predominantly White-led) academic institutions, in which they are a subcontracted organization that can provide traditional healing services. This partnership can be beneficial: ICBOs can attain higher amounts of funding to provide cultural psychosocial services and contribute to the growing evidence of their efficacy. Subsequently, funders increasingly accept traditional healing activities as direct costs.

However, funders still prefer that traditional healing as a direct service be coupled with or adapted from Western evidence-based practices grounded in Western clinical or psychological science. The necessary resources, time, and effort (including training and credentialed staff) to provide or adapt such practices can exceed ICBOs’ capacity. ICBOs therefore are less able to independently attain such grants, and they continue to depend on subcontracted partnerships (appendix exhibit 2b, loop B).^27^ Unfortunately, there is little opportunity for ICBOs to build their own capacity and infrastructure beyond a subcontracted relationship, given the low amount of indirect funding from these contracts. This practice allows for larger White-led organizations to remain at a higher level of power as “brokers” of the capital and capacity required to continue sustainability for ICBOs to provide communities with traditional healing services (appendix exhibit 2b, loops C and D).^27^

### WITHIN COMMUNITY, BETWEEN-CBO COMPETITION

We identified several reinforcing feedback loops between ICBOs and White-led CBOs that reduce the sustainability of ICBOs within communities. These loops involve competing for grants and clients, the funding policies (single-issue, grant-funded services and service provision–based success metrics) fueling this competition, and the “catch-22” that ICBOs experience with subcontract relationships that disproportionately benefit White-led CBOs (appendix exhibit 2c).^27^ Year after year, new ICBOs will emerge to meet the enduring needs for Indigenous people. Subsequently, ICBOs often compete with each other over the limited pool of available funds to attain the large number of grants they require to be sustainable (appendix exhibit 2c, loop A).^27^ Funding competition is reinforced by the high prevalence of grants that fund direct services for single issues. Despite increased grants for Indigenous needs, the number of ICBOs that can attain such grants is limited because of the staff and resources required for addressing the specific issue that each grant funds (see exhibit 1 for examples). The resulting limited ICBO sustainability stagnates ICBOs’ provision of care and the ability for ICBOs to collectively satisfy the needs of their communities.

Grants developed to address Indigenous needs are also attractive for White-led organizations (appendix exhibit 2c, loop B.)^27^ Although this can exacerbate funding competition, ICBOs can form subcontract relationships with higher-capacity, White-led organizations that are able to procure larger grants, mitigating some funding competition–related challenges. ICBOs can benefit from this relationship, as they can receive more funding and use the larger organization’s capacity to more efficiently provide culturally based psychosocial care. However, subcontracts involve a hierarchy in which the larger organization maintains funding control, and ICBOs experience disproportionate drawbacks. Subcontracts provide little support to help ICBOs grow their own capacity and infrastructure for continuing service provision after the grant ends. Continued underdevelopment of ICBOs’ capacity maintains their dependence on subcontracts. This dependence is also emphasized during the grant project itself, as larger organizations control the disbursement of funds to subcontracted partners. ICBOs are therefore more vulnerable to issues such as funding delays, reducing their ability to provide effective services even in a subcontracted relationship. Subcontracted relationships also disproportionately benefit the larger, White-led organizations that subcontract ICBOs. They are able to use the larger amount of indirect funds from grants (through a higher indirect cost rate) to more efficiently maintain their larger infrastructure as a result of higher funding stream diversity (providing more reimbursable services to a larger population of non-Indigenous clients).

Subcontract partnerships allow White-led CBOs to demonstrate their own success for future funding through the subcontracted ICBO’s efforts (appendix exhibit 2c, loop C).^27^ Funders often use service provision as a metric of project success for single-issue service-provision grants (and an indicator of future funding). The relatively small population of Indigenous people as potential clients shrinks further when single-issue grant parameters result in a narrow pool of eligible clients. ICBOs therefore compete with each other to provide services for a limited group of community members, reducing their ability to demonstrate grant success for future funding. Subcontracted partnerships can also reduce the potential threat of client competition that ICBOs experience from White-led service providers. When not working together, White-led organizations can see ICBOs as competition in the provision of services for Indigenous community members. The strength of the White-led service provider, and its threat as a competitor for clients, reinforces the incentive for ICBOs to remain in inequitable subcontract relationships.

### WITHIN-COMMUNITY, BETWEEN-CBO NETWORK COLLABORATION

We identified three key reinforcing loops and one balancing loop related to within-community CBO collaboration. These loops highlight the risks for ICBO service duplication, which reduces ICBOs’ sustainability and the ability for ICBOs to collaborate with each other, forcing dependence on existing networks of collaborative care led by White CBOs (appendix exhibit 2d).^27^ The myriad issues faced in Indigenous communities, combined with the narrow specificity for what grants can fund, result in service duplication (appendix exhibit 2d, loop A).^27^ In turn, a churn of unsustainable ICBOs with limited capacity for community engagement outside of direct service provision limits community knowledge of existing services. The combination of needing a large number of services to address all individual and community needs and having little knowledge of the community’s current service landscape further increases the risk that new ICBOs will duplicate services (appendix exhibit 2d, loop B).^27^ Together, this competition reduces community-level trust in services and ICBOs’ ability to form a collaborative network.

Navigating a large number of duplicated and siloed services (for example, connecting with other service providers to provide and receive referrals) can be burdensome for ICBOs. However, this navigation is critical for ICBOs to ensure that Indigenous community members receive the multiple services they need (appendix exhibit 2d, loops C and D)^27^ and to sustain operations themselves. Thus, ICBOs often depend on existing (White-led) networks of comprehensive care. Although this improves the existing network’s ability to provide effective services for Indigenous community members, it also further reduces the ability of ICBOs to form their own networks of care.

## Discussion

Funding policies for the grants that ICBOs attain are incompatible with their needs to thrive. As a result, ICBOs maintain gaps in infrastructure; foster competition and nonsustainable resources; and depend on larger, White-led organizations. We extend the literature on the challenges posed by funding policies faced by community-based organizations to provide psychosocial care^19,31^ with a focus on a seldom-discussed but critical facet of Indigenous health. Our findings highlight how these challenges inequitably leave ICBOs vulnerable to falling into capability traps that require them to attain lower funding at the expense of sustainable growth.^15,18^ Specifically, the funding policies discussed here hinder ICBOs’ ability to collaboratively provide self-determined psychosocial care (that is, care without the constraints of specific direct services or requirements to use non-Indigenous evidence-based practices).

Increasing funding for the IHS and psychosocial care for Indigenous populations is necessary for Indigenous health investment. However, the policies for this funding are still grounded in an oppressive framework in which Indigenous health is overly regulated.^9,15^ Instead of removing barriers of structural racism, increasing funding without addressing funding policies ensures that these barriers maintain their resilience.^20^ Here, we discuss three broad areas of funding policy to address and suggested strategies for addressing them. More research is required to evaluate the nuances related to different funding types (for example, private, public, federal, or local).

### INDIRECT AND DIRECT COSTS

First, funders must address indirect cost rates and allowable direct service expenditure regulations. The subcontracts and small grants that ICBOs typically attain will inadequately support building capacity and infrastructure. Increasing indirect cost rates and providing more grants specifically for capacity and infrastructure building have been previously suggested strategies.^32^ ICBOs may also benefit from funders redefining direct services and allowable expenditures to accurately reflect what it costs ICBOs to provide effective care. This redefinition would allow for traditional healing as part of direct services and would include costs associated with traditional healing (including food, materials for ceremonies, and compensation for culturally appropriate ceremony leaders). Accounting for the time, effort, and resources needed to provide direct services effectively, based on contexts of community and Indigenous historical trauma,^31^ will also be necessary. For example, building authentic relationships with community members before providing any services is a critical value in Indigenous care that is much needed in contexts of trauma and mistrust.^14^ Relationship-building costs are often ignored when “effort” is being defined for service provision.

Indirect and direct costs need to be modified for subcontract partnerships. First, restructuring the hierarchical way in which funds are disbursed (more direct disbursement to both larger and subcontract organization) will reduce power imbalances. Providing subcontracted ICBOs with additional funds to build sustainable independent service provision infrastructure (potentially in part through the larger organizations’ cost sharing) can be coupled with support for relationship building between organizations. Building authentic relationships beyond a business transaction could include bidirectional knowledge sharing. Larger White-led organizations can provide training and resources for writing, managing, and carrying out successfully funded projects. ICBOs could provide education and consultation on culturally appropriate practices.

### COMPETITION AND COLLABORATION

A second area for change is for funders to address how ICBO competition reduces sustainability and collaboration. One approach could be for public or larger private funders to develop a multi-ICBO collaborative care grant. Similar mechanisms do exist, such as the Substance Abuse and Mental Health Services Administration’s Circles of Care mechanism.^33^ However, a new mechanism could enhance collaboration by equitably allocating funds across multiple organizations, instead of funding one organization.

Grants could also shift from focusing on individual ICBOs’ psychosocial care provision to restructuring how ICBOs collectively provide, advocate for, and sustain care. Coalitions could redefine how care can be addressed holistically, encouraging inclusive participation beyond service-providing ICBOs, including Indigenous stakeholders from varied areas in health-related policy, education, outreach, and research. Importantly, these coalitions could develop strategies to increase the ability for ICBOs and collaborators to have alternative funding streams for providing care. For example, advocating for traditional healing care reimbursement beyond culturally adapted evidence-based practice would be a clear strategy to reduce external grant dependence. The Maniilaq Social Medicine Program is an example of an Indigenous network that holistically addresses a range of social and health needs. Activists, academics, clinicians, and policy makers partner to provide services, research, training, education, and policy development to strengthen the capacity and improve the availability, accessibility, and quality of Indigenous-led care.^34^

Given participating organizations’ low capacity, transformative collaborations may require a two-phase mechanism that funds planning and initial action. Start-up funding would allow collaborating organizations to better focus on developing their plans, knowing that they would not have to simultaneously search for additional funding sources to implement plans. This approach may require funders to provide larger, but fewer, grants. Although this would reduce the number of ICBOs that may receive funding, stronger funding support can increase awardees’ success (reducing the funding capability trap effect). Some private funding organizations have started to use this strategy^35^ to better meet awarded organizations’ needs.

### REDUCING BARRIERS

A third area for change will be for funders to reduce barriers to grant seeking and management. ICBO leaders often lack access to the resources needed to successfully attain and implement grants. Grant “social capital” includes knowledge of how to find potential funding initiatives, write successful applications, and manage grants and projects.^15^ ICBOs would benefit from funding agencies tailoring application and regulatory processes to better match the size of the grant or organization grantee.^32^ ICBOs and other Indigenous organizations can struggle to comply with grant expectations, such as conducting an evaluation or sustainability plan after grants are awarded.^36^ Therefore, funding agencies could increase ICBOs’ access to education, training, and low-cost resources (for example, grant management software or funding opportunity email discussion lists) for all aspects of the grant process from application to project end.

## Conclusion

Reliance on CBOs to provide psychosocial care and social welfare continues to grow. As this has become a popular strategy to provide community and culturally grounded care for minoritized communities,^12^ understanding how structural racism shapes organizational efficacy to provide care is critical. Unique contexts for Indigenous communities, characterized by both oppression and cultural strength, require distinct consideration. The role of funding in self-determination of health has been discussed primarily in relation to Tribal and IHS contexts.^2^ Even in areas where the IHS is physically available, psychosocial care accessibility remains challenging.^14^ Investing in Indigenous self-determined health requires changing the investment process from top-down provisional structures to bottom-up growth and empowerment of Indigenous communities.

## Supplementary Material

supplemental materials

## Figures and Tables

**EXHIBIT 1:** Overview and challenges related to key funding policies affecting Indigenous community-based organizations (ICBOs)

Policies	Definitions	Challenges
Regulations on direct service expenditures	Regulations that define what can considered as direct costs (expenditures directly related to carrying out grant-funded project activities, such as salaries or project materials) in a grant.	Service-specific rules govern the permissible direct cost outlays in grants, prescribing the budget share for distinct activities such as staff or service provision. Justifying certain expenditures critical to Indigenous psychosocial care, such as food, ceremonial items, or relationship-building efforts, as direct costs is often challenging.
Indirect cost rates	Grant- or funder-specific guidelines determining the proportion of indirect costs (expenses for general overhead that is not related to a specific project, which includes broader infrastructure and capacity building) that an organization needs to carry out its objectives.	Larger organizations often negotiate for indirect cost rates higher than the standard 10% rate that is used for many small organizations. Negotiated rates are based on their structure rather than individual projects. Private funders typically set a fixed rate that is lower than many negotiated rates but higher than the 10% minimum, often around 15%.
Funder requirements for grant compliance	The amount and types of information required for grantees to collect and report to funding agencies over the duration of the grant to monitor project progress and compliance.	Grantees must provide extensive details on budget, organization finances, and performance metrics such as service reach and goal progress, necessitating data collection and reporting. The obligations vary little based on grant size, and differing mandates from various funders add complexity.
Single-issue, direct-service focus	The tendency for psychosocial grant mechanisms to have narrow parameters for what types of psychosocial needs grantees can address and the types of strategies they can use to address them.	Grants typically target specific psychosocial conditions or demographics (such as specific substances in substance use treatment grants) and focus on certain prevention-intervention stages (for example, primary prevention only), which can conflict with Indigenous communities’ needs for comprehensive care due to high comorbidity rates and holistic health perspectives.
Prioritization of Western evidence-based practices	Funding-mechanism requirements to use specific evidence-based practices for providing psychosocial care.	Grants often require evidence-based care justification, promoting specific approaches or adherence to best practices. This is well justified but can burden ICBOs lacking the needed infrastructure or credentialed staff. Moreover, generic practices are less effective for Indigenous communities than culturally adapted ones, requiring further resources and expertise that ICBOs may lack.
Service provision as success metric	Using engagement and provision numbers (number of clients or times resources are provided) as a metric for evaluating progress and success of grants.	Service provision grants usually necessitate reporting on service numbers or client engagement against preset goals, a key evaluation metric for funders. While necessary, this might not reflect an ICBO’s success in addressing disparities and promoting healing, especially in regions where Indigenous populations are a smaller minority or in rural areas.

**SOURCE** Data collected from participatory modeling.
