# Supplementary material for: How Funding Policy Maintains Structural Inequity Within Indigenous Community-Based Organizations
Source: Health Aff (Millwood). Author manuscript; Available in PMC 2025 Sep 25. (PMC12462777; doi:10.1377/hlthaff.2023.00483)

Appendix

Appendix 1
Supplementary Description of the Study

Sample

The sample for this study is composed of 148 participants in a Northern Great Plains small metro who have a variety of personal or professional experience within broad areas of Indigenous health, intimate partner violence, alcohol or substance use/alcohol and substance-exposed pregnancy, and perinatal health. Overall, this included individuals who had personal or professional experience in alcohol and substance use treatment (for pregnant women especially), domestic violence and domestic violence shelters, policing and justice, public defense and legal systems, re-entry, drug and mental health courts, child welfare services, nonprofit and public social services, nursing, counseling, and Indigenous advocacy and health. The majority of participants for the entire sample was female, and approximately 50% Indigenous; there were clear disparities in race/ethnicity differences for people who had *personal* experience (Indigenous) and people in *professional* roles (non-Indigenous), which was reflective of the overarching power dynamics within the specific site community. We focused on outreach to Indigenous community members who have experience in both Indigenous-led and non-Indigenous-led organizations providing care for one of the multiple issues. As the study progressed, especially for the refinement sessions (see below), our sample became predominantly Indigenous, involving participants who had both personal and professional experience with the issues of interest. Furthermore, consistent discussion of funding and organizational capacity among many of the sessions prompted us to recruit participants that specifically had experience in grant funding for nonprofit social service organizations, both non-Indigenous and Indigenous.

Data and Procedures

We created a protocol that allowed for individual aspects of the model to be explored through short duration model building sessions, which was important given the substantial barriers for larger groups and longer-term modeling sessions (e.g., time constraints, mistrust, and privacy concerns between people with personal and professional experiences in the same groups). Therefore, modeling sessions focused on specific aspects of the system, or areas of expertise (e.g., personal experience with domestic violence, professional perinatal health experience). We developed a script that included variable elicitation and causal loop diagramming, which lasted 1 ½ hours and could be facilitated in person or remotely with video-conferencing applications in either small groups or with individuals. Individual sessions were particularly important for participants who did not feel comfortable or safe discussing personal experiences, thoughts, or feelings with others. Participants only participated in one session. Scripts are structured activities commonly used in community-based system dynamics studies, in which participants engage in building the model through eliciting variables, connecting them, and identifying loops.

Initial sessions started from a “blank slate”, in which participants listed 3 causes and 3 effects of alcohol/substance misuse, intimate partner violence, and alcohol/substance-exposed pregnancy. Variables were then voted upon (if in small groups, e.g., the “dot script”). The modeler/co-facilitator then drew an initial causal loop diagram using the most popularly voted upon variables, either on a whiteboard visible to in-person groups/individuals or using Vensim modeling software and sharing their screen with participants. This diagram was then discussed, as participants considered what new variables should be added and how connections between variables should be formed, either in a focus group/talking circle or interview format. At later stages, when specific areas of the model required more information, a “starter causal loop diagram” was presented to the participants, in one or two variables relevant to the specific area of

needed information (e.g., substance use treatment access, reproductive health, and birth control) were included, and participants then provided insight and proceeded with the same causal loop diagramming process as conducted in earlier sessions.

Model sessions were hosted at a variety of locales, including in organizations, in a local library, and virtually (especially as data collection continued during 2020/2021). Data included the drawn models and notes taken during the conversation by research staff, although later models included audio recording that was transcribed to reduce the burden of notetaking. Overall, there were 25 subject-specific participatory modeling sessions, which focused on specific aspects of the overarching system for approximately 120 participants attending the sessions.

Individual models were then aggregated to create a consolidated full model that could be further explored in a series of more intensive modeling critique sessions. This aggregation was done by research staff using a grounded-theory, synthesis approach to identify variables thematically. The consolidated model included 149 variables in 8 sub-models that cut across all sections. We then hosted a series of 5 “model refinement” sessions in which small groups of 8 – 10 participants (28 participants total over all sessions, 80% female, 90% Indigenous) discussed parts of the consolidated model to validate, critique, or improve. Participants once again were those who had personal or professional experience in the subject areas. Similar to the way in which the initial model sessions started to shift towards the allocation of resources and funding, substantial time during these sessions was devoted to discussing the way in which addressing the issues required substantial changes to the way in which funding for care was structured.

Relevance to the Current Model

The models for this paper are based upon specific aspects of the broader collection of models that focused on funding, organizational capacity and operations, and relevant specific areas of structural and systemic racism experienced by Indigenous community members. Therefore, the specific “issue of focus” of the project (alcohol/substance use, intimate partner violence, unintended pregnancy/alcohol, and substance-exposed pregnancy) is not included. Rather, we borrow heavily from individual models that focus on funding and provision of services, and the broader discussions and transcripts from the refinement models focusing on allocation of services and service provision.

Additional Information

The models were also shaped through iterative discussions and the collaborative partnerships between the researchers and community member co-authors, all of whom have long-term experience in working with and leading ICBO efforts to provide culturally-grounded psychosocial care, and experience in broader collaborations between Indigenous and non-Indigenous public and private institutions and organizations. These partnerships are grounded in a community-based participatory framework in which the primary investigator collaborates with partners on multiple efforts including grant writing, evaluation, and research driven by community partner needs and interests.

The guideline in **Appendix Exhibit 1** provides details about the definitions of the symbols shown in **Appendix Exhibit 2**.

**Appendix Exhibit Table 1:** Overview of causal loop diagram notations used in this document	
**Symbol**	**Definition**
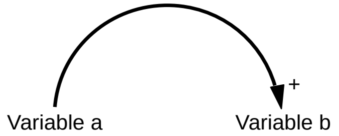	The positive sign represents an increase (decrease) in variable ‘a’ leads to an increase (decrease) in variable ‘b’ – that is a change in the same direction.
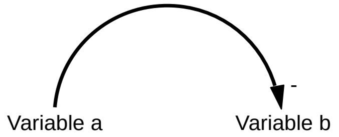	The negative sign represents an increase (decrease) in variable ‘a’ leads to a decrease (increase) in variable ‘b’ – that is a change in the opposite direction.
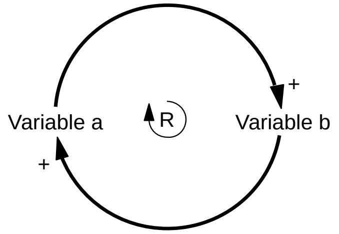	“R” represents a Reinforcing loop whereby an initial increase in variable ‘a’ causes an increase in variable ‘b’, leading to a consequent increase in ‘a’. Also, a decrease in ‘a’ causes a decrease in ‘b’, leading to a consequent decrease in ‘a’. A reinforcing loop is a part of the system that results in subsequent exponential change – either growth or decay.
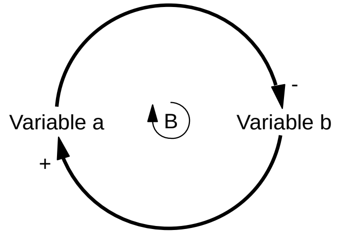	“B” represents a Balancing loop whereby an initial increase in variable ‘a’ causes a decrease in variable ‘b’, leading to a consequent decrease in ‘a’. Also, a decrease in ‘a’ causes an increase in ‘b’, leading to a consequent increase in ‘a’. A balancing loop is a part of the system that ‘self-limits’, ‘slows’, or ‘brings towards equilibrium.’

In **Appendix Exhibits 2a-d**, we present individual loops and provide explanations. Funding policies discussed in the manuscript are bolded in green.

**Appendix Exhibit 2a**: Selected feedback loops relevant to funding cycle capability traps for Indigenous Community-based Organizations (ICBOs)		
**Summary**	**Visualization of Loop**	**Explanation of Loop**
**Loop A** (Reinforcing) ICBOs struggling to close operational gaps while grant seeking	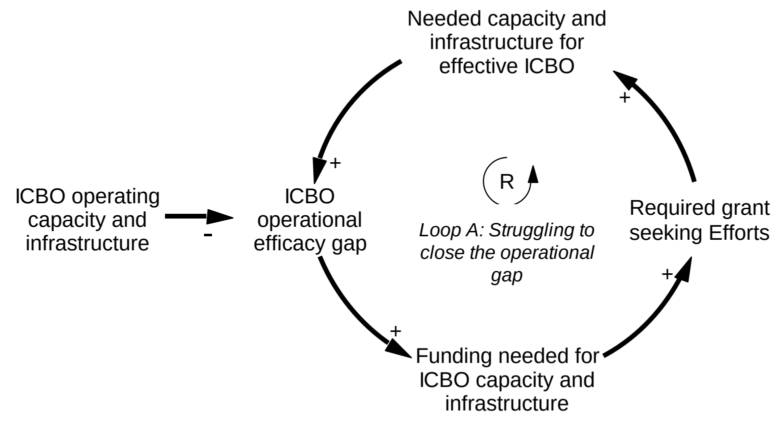	ICBOs often start with large *operational efficacy gaps*, which is the difference between their realized *ICBO capacity and infrastructure* and the *capacity and infrastructure required* for effective operational functioning. The larger the gap, the more *funding the ICBO will need* to support capacity and infrastructure, which fuels an increase in the level of *effort required to seek grants* and funding. Grant seeking consumes the existing capacity of ICBOs (e.g., grant seeking, writing, preparing), and thus they will need even more capacity and infrastructure to be effective, ultimately increasing the operational efficacy gap they are trying to reduce.
**Loop B** (Reinforcing) ICBOs losing capacity and infrastructure due to the size of grants they are eligible for and able to actually obtain	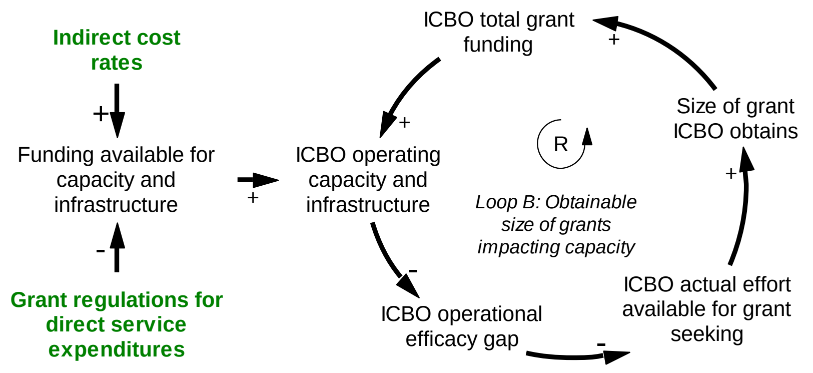	Driven by a strict *regulations that use narrow definitions of direct service expenditures* and low *indirect cost rates* for indirect funds, grants provide ICBOs with a limited amount of *funding to invest in their capacity and infrastructure*. Lower capacity increases their *operational efficacy gap*, which reduces the *effort ICBOs have available for grant seeking*. Lower effort available for grant seeking will reduce the *size of grants* (amount of funding in grants) that ICBOs can actually obtain. Therefore, they will have lower *total grant funding*, which reduces their overall *capacity and infrastructure*.

<table>
<tr>
<td>Loop C
(reinforcing)
ICBOs losing capacity and infrastructure through seeking a larger number of smaller-sized grants</td>
<td>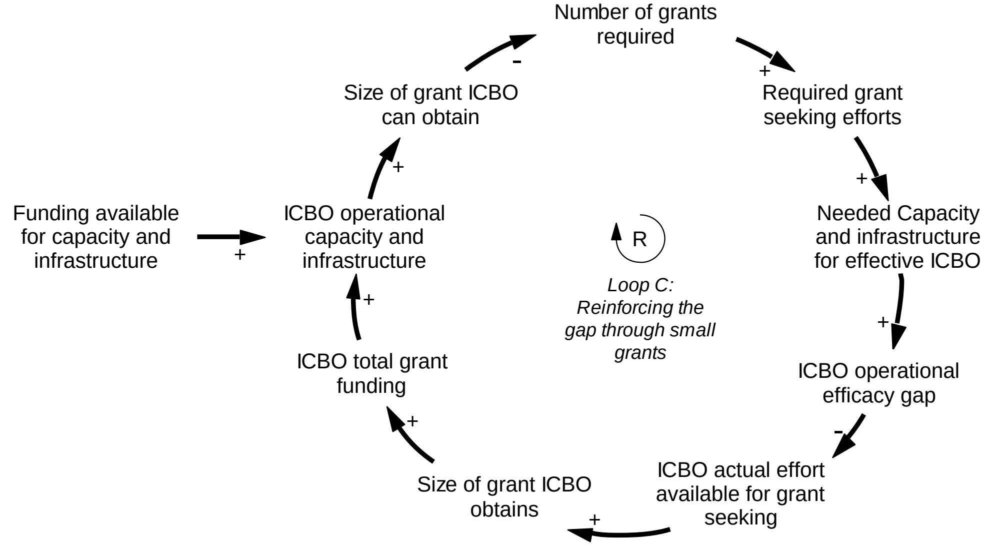
</td>
<td>As the size of grants that ICBOs can obtain decreases, they will need to attain a larger number of grants to be a sustainable and effective organization. Seeking a higher number of grants will tax current infrastructure, increasing the effort and operational infrastructure they require. This will increase their operational efficacy gap, feeding into feedback loop B, described above, again driving reductions in the actual ICBO capacity and infrastructure.</td>
</tr>
<tr>
<td>Loop D
(Balancing)
Size of grants ICBOs receive for just enough to survive

Loop E
(Balancing)
Number of grants ICBOs receive for just enough to survive</td>
<td>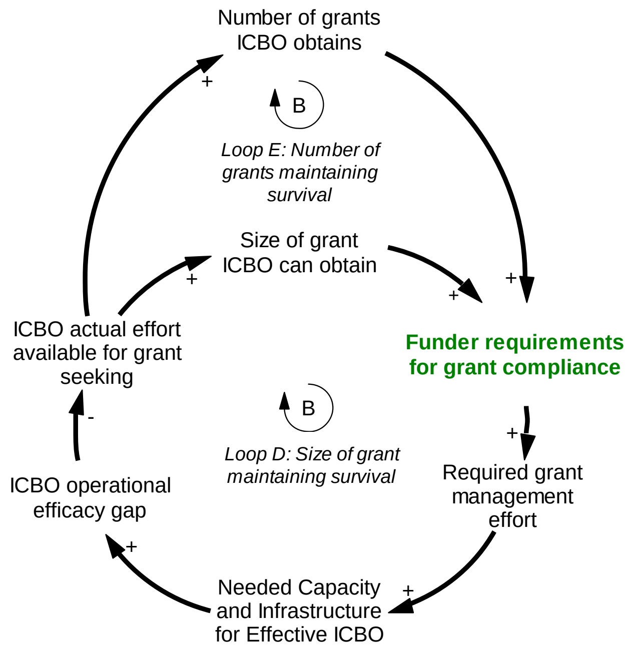
</td>
<td>As ICBO's effort to seek grants decreases, the size of grants they obtain also decreases. Smaller grants can have lower regulatory and compliance requirements for grant management, therefore reducing the effort required for grant compliance and regulation, and the infrastructure required for an effective ICBO. This will lessen the operational efficacy gap, thereby increasing the effort available for grant seeking. In turn, this can increase the allowable effort for grant seeking, which can increase the size of the grants that ICBOs can obtain. However, obtaining larger grants increases the effort ICBOs require for grant compliance and regulation, and their operational efficacy gap, once again reducing effort available for grant seeking.
The same feedback pathway operates through the number of grants the ICBO obtains. These two pathways create a balancing force that, in conjunction with a consistent need for more grants, holds the ICBO in a cycle of striving for larger and more grants but holding them below a threshold where they essentially obtain, “just enough to survive”.</td>
</tr>
</table>

Appendix Exhibit 2b: Selected feedback loops relevant to the role of evidence-based practices in creating capability traps for ICBOs

Summary	Visualization of Loop	Explanation of Loop
**Loop A** (Balancing) Advocacy for culturally appropriate services for Indigenous communities improves limited partnership of white organizations and ICBOs	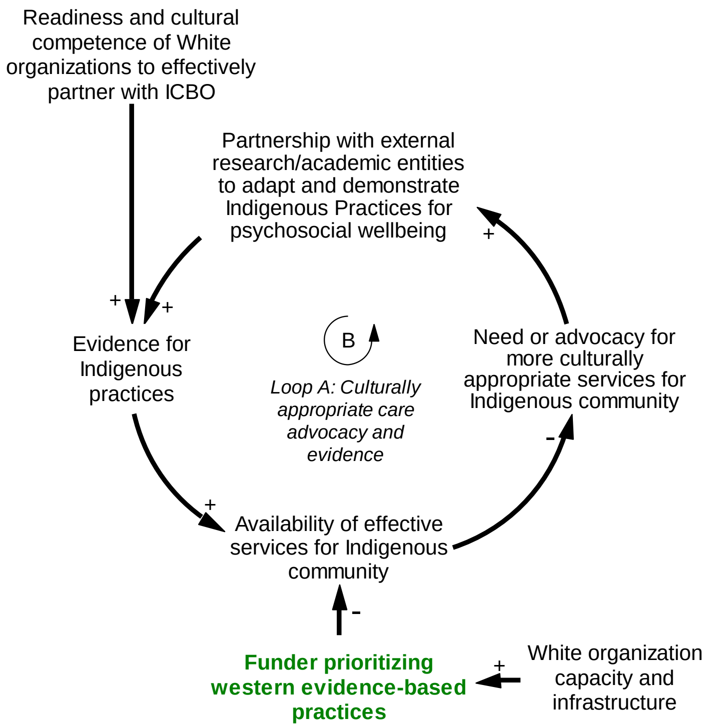	White organizations have historically had higher *capacity and infrastructure*, which had led to greater *reliance on western evidence-based practices* and lessening the *availability of effective services for Indigenous communities*. This has increased the *need and call for more culturally appropriate services for Indigenous communities* and an increase in *partnerships between external research/academic entities to adapt practices* and ICBOs (e.g., predominantly white academic research organizations and ICBOs). These partnerships do have the potential to improve *evidence for Indigenous practice*, though other factors are important in this association, such as the *readiness and cultural competence of the white organization* and their personnel to work with ICBOs effectively. There is also a concern that as evidence is created, there could be a subsequent reduction in the partnerships between white organizations and ICBOs, without the full realization of increasing ICBOs' capacity and infrastructure to provide the needed services.

**Loop B**<br>(Reinforcing)<br>Success to successful white organizations	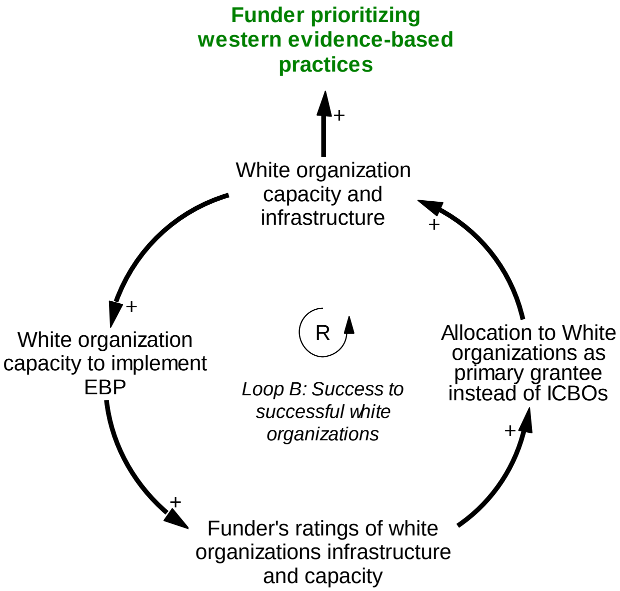	It is important to underscore how traditionally higher rates of funding has increased *white organizations' capacity and infrastructure* compared to ICBOs. These organizations therefore have higher *capacity to implement evidence-based practices*, particularly as they have been created using western philosophies and epistemologies of research and knowledge creation. This also leads to the *funder's rating* of white organizations as having higher infrastructure and capacity, and thus funds are often *allocated to white organizations,* completing a reinforcing cycle. This is an archetypical dynamic of success to the successful.
**Loop C**<br>(Reinforcing)<br>Subcontracts for evidence-based practices reducing ICBO capacity<br><br>**Loop D**<br>(Reinforcing)<br>Subcontracts for evidence-based practices maintaining ICBO need to subcontract	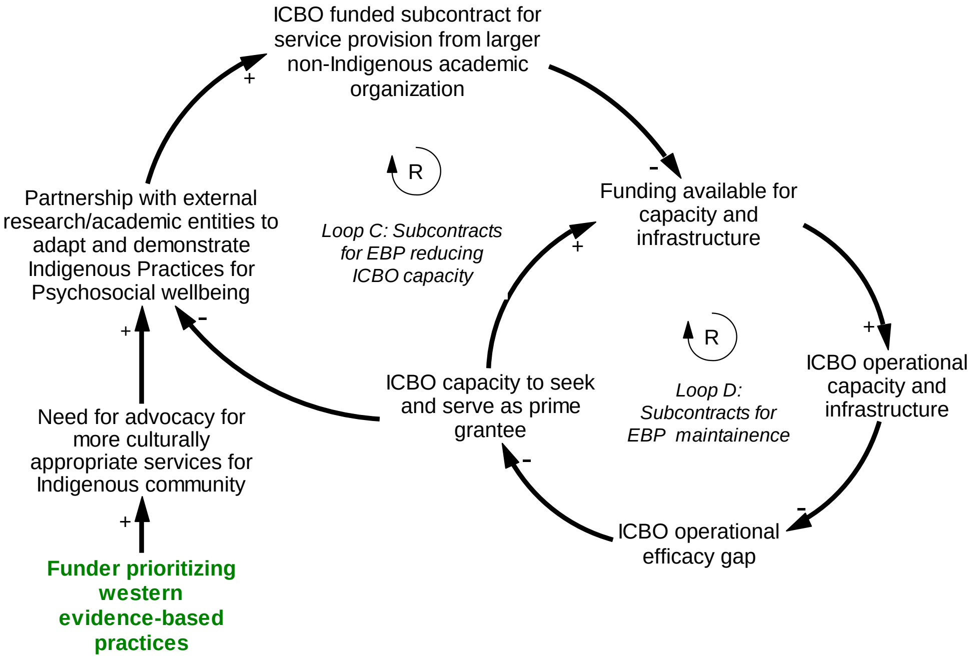	As white organizations are successful, they work with *ICBOs as subcontracting agencies* in order to help fulfill the needs for *more culturally appropriate services for Indigenous communities*. This, however, leads to a limited proportion *of funding available for capacity and infrastructure* (as subcontracts typically provide a low amount of indirect funding based on the specified amount of funds for the contracted service), which then further reduces *grant funding and capacity and infrastructure*, increasing the *operational efficacy gaps* and lessening *ICBO's ability to seek and serve as the prime grantees* of larger grants and funding sources, thereby maintaining low *ICBO capacity to seek and serve as prime grantee*

<table>
<tr><td colspan="3">Appendix Exhibit 2c: Selected feedback loops relevant to the role of funding and client competition challenges for ICBOs</td></tr>
<tr><td>Summary</td><td>Visualization of Loop</td><td>Explanation of Loop</td></tr>
<tr><td>Loop A
(Balancing)
Resistance to change through ICBO funding competition</td><td>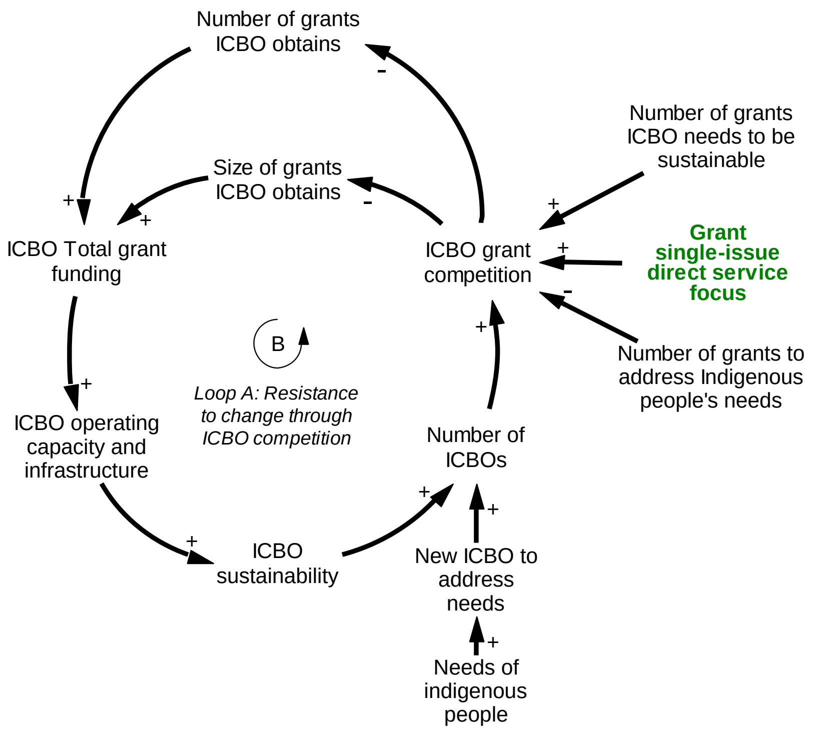
</td><td>The overall competition for funding among ICBOs impacts ICBO sustainability on a community level. Specifically, new ICBOs will be created to address the high number of Indigenous People with unmet needs. The more ICBOs in a community, the more these ICBOs will have to compete with each other for the limited grants available, especially as they tailor their work to fit the narrow grant funding criteria through grants' focus on single issues for direct services. This competition decreases the average ICBOs grant funding, capacity and infrastructure, and sustainability, thereby reducing the number of ICBOs. The reduction in ICBOs decreases competition between ICBOs again cycling through the same pathway will improve ICBO sustainability and decrease the number of new ICBOs. However, in the absence of other factors (increased numbers and sizes of grants) the cycle continues, and ICBOs are forced to resist efforts for changing this cycle, continuing to compete for limited funding.</td></tr>
</table>

**Loop B**<br>(Balancing)<br>Resistance to change through unmet needs	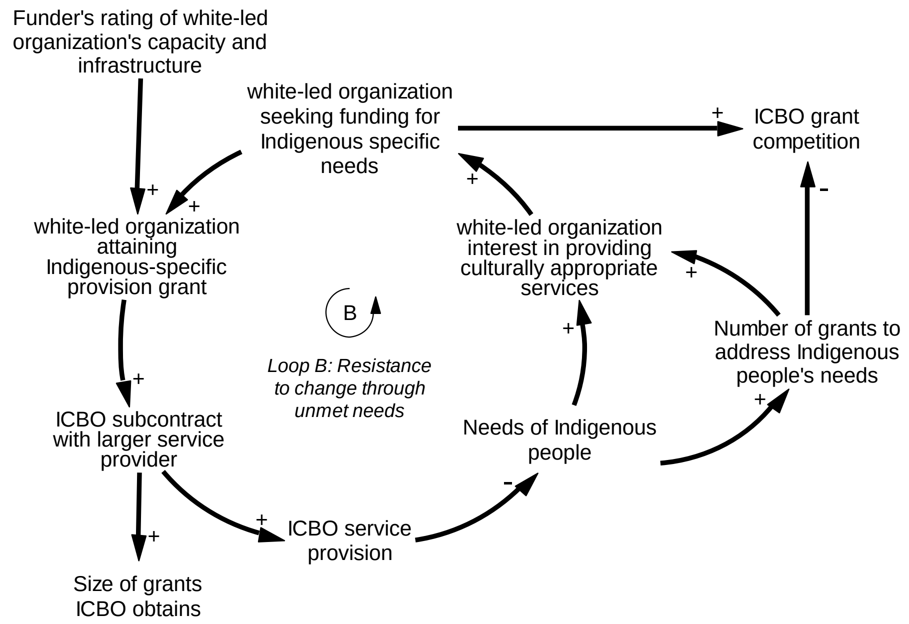 	There is a resistance to change that keeps white organizations more successful while never fully addressing Indigenous People's unmet needs. Specifically, to address growth in the number of *Indigenous People with increasing unmet psychosocial needs* organizations and institutions will increase the *number of grants to address these needs.* As funding to address Indigenous Peoples needs increases, *white organization's interest* in these funds, by providing culturally appropriate services will increase as well. More white organizations attaining these funds will shrink the pool of grants available, *increasing the competition between ICBOs for the remaining gran*ts (further leading into previously discussed issues to increase ICBO capacity). Further, as described in Exhibit 2, the grant funding is more likely to be *allocated to the white organization* increasing their *use of ICBOs as subcontracting agencies,* again feeding into the reduced *capacity of ICBOs to obtain large grants*. Positively, this increase in ICBO as subcontracting can reduce the number of Indigenous People with unmet psychosocial needs. However, also leads to continued resistant cycles whereby ICBOs limited capacity is reinforced, ICBOs are not used as subcontracting agencies, and *the Indigenous People's needs are increased once again*.

**Loop C**<br>(Balancing)<br>Resistance to change due to single-issue service competition	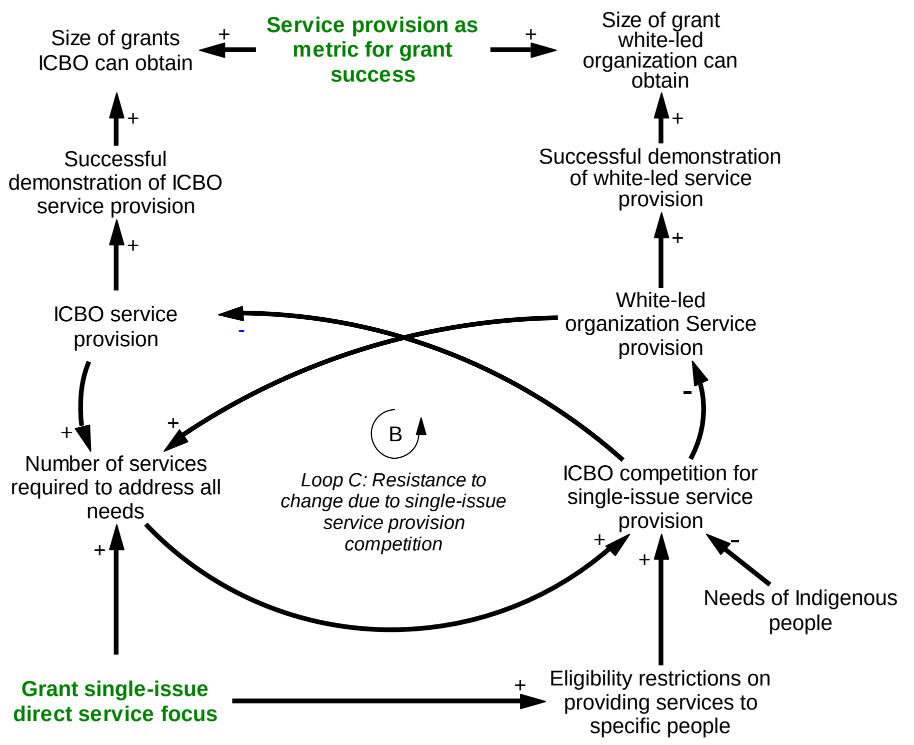	Funder's *emphasis on single issues for direct services* also feeds into a resistant cycle. Competition generated through single-issue grants creates a *level of competition between all organizations*. As service provision is an indicator of success, this competition is dangerous for the longevity of the organization. Further, competition can reduce all organizations from being able to *provide efficient services.* This can reduce the *number of services addressing single-issue needs*, which in turn reduces *competition just enough to allow for organizations to provide services*. However, over time, this will lead once again *to increased competition* as enduring unmet needs for Indigenous People will give rise to new ICBOs, and therefore new competitors. Thus, this is a vicious trap all the while competition feeds into problematic cycles of keeping ICBOs from improving their capacity while white organizations continue to grow.

<table>
<tr><td colspan="3">Appendix Exhibit 2d: Selected feedback loops relevant to the role of organizational network competition and collaboration challenges for ICBOs</td></tr>
<tr><td>Summary</td><td>Visualization of Loop</td><td>Explanation of Loop</td></tr>
<tr><td>Loop A
(Reinforcing)
Duplication of services and competition</td><td>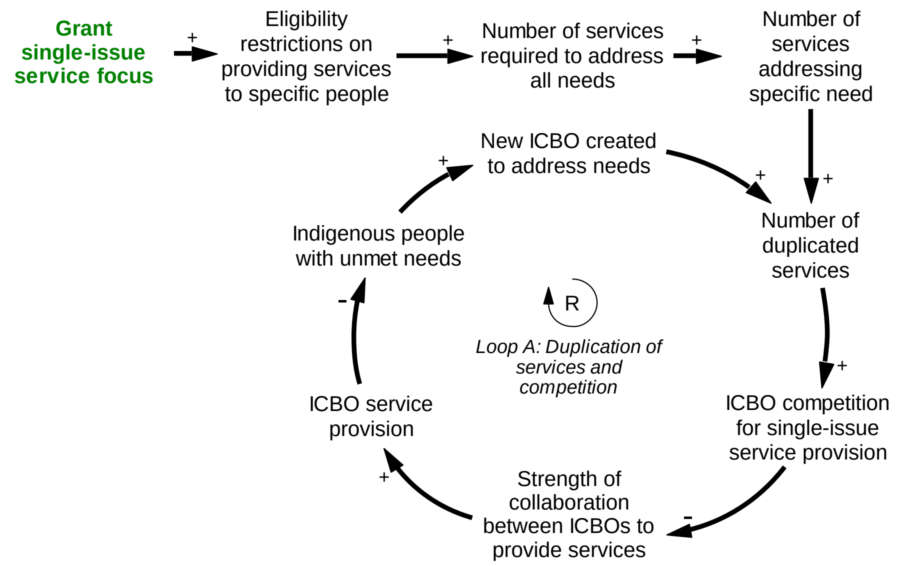
</td><td>Funding emphasis on single-issue direct services increases the likelihood of service duplication and competition between ICBOs, reducing the level of coordinated care and ultimately meeting the needs of the population. Specifically, as increased restrictions are placed on who can receive the single-issue services, the number of services that are created to address that specific need (tailoring services for the grant), leading to an increase in duplicative services. Duplication in services increases competition, reduces collaboration of ICBOs, which reduces the provision of services and increases unmet needs. This leads to an increase in new ICBOs to address needs and, hence, more duplicated services.</td></tr>
<tr><td>Loop B
(Reinforcing)
Competition reduces ICBO sustainability.</td><td>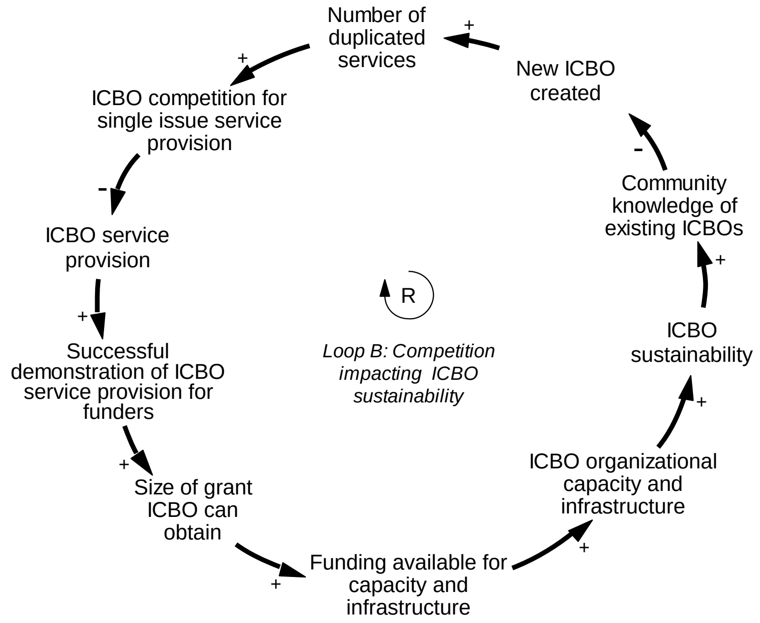
</td><td>The increased competition to provide specific service decreases ICBO service provision and successful demonstration of ICBO service provision for funders. Without demonstration of success, ICBOs can only obtain smaller grant sizes, which results in reduced funding, organizational capacity and infrastructure, and sustainability. As ICBOs are not sustained, there is limited knowledge of what organizations (and services) exist, leading to new ICBOs created to address an assumed gap in need, duplicating services, once again, increasing the competition among organizations to provide single-issue services for which funding is available.</td></tr>
</table>

**Loop C** (Balancing) ICBOs' forced dependence on existing networks for subcontracts / referrals<br><br>**Loop D** (Reinforcing) Network reliance reduces ICBO ability to create their own network of service and care provision	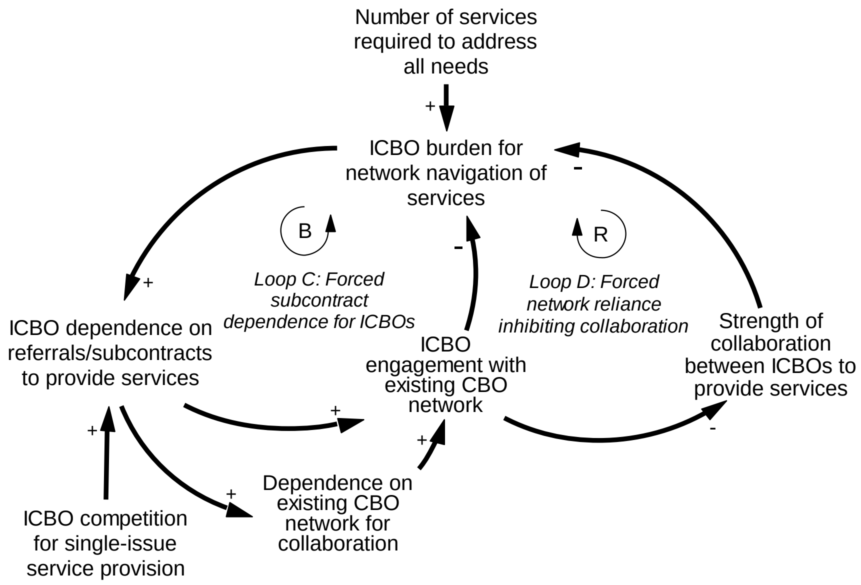	Requiring a large *number of services to address all needs* substantially increases the *burden of ICBOs to navigate this large network* of multiple and siloed services. This burden increases ICBOs' *dependence on the existing network*, (which is composed of predominantly white organizations), as they need to collaborate with this network to help with navigation, and as they *are dependent upon referrals and subcontracts* that are attained through this network**.** However, higher *engagement with the existing network* also reduces the *strength of collaboration between ICBOs*. As the strength of cross-ICBO networks is weakened, they become a poor alternative source for reducing *network navigation burden* and as a source for referrals and contracts. This, in turn, continues to reinforce the need for ICBOs to *depend on the existing collaborative networks.*

Appendix Exhibit 3: Full detailed model for ICBO funding and mechanisms of structural racism

Variables depicted with a bracketed outline in gray text signify their location in another section of the model, helping to prevent crossing causal links.

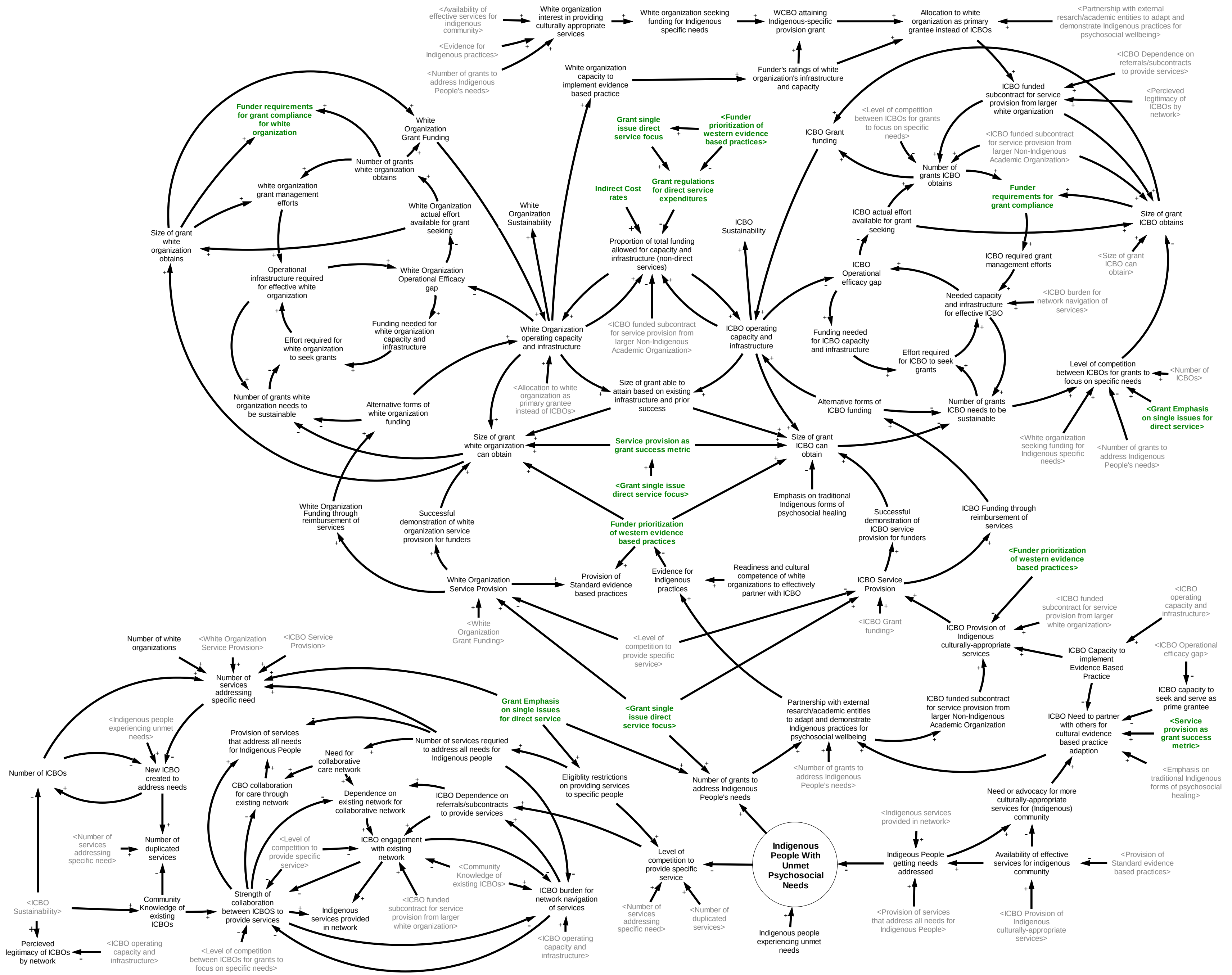

Supplement: supplemental materials [file NIHMS2112303-supplement-supplemental_materials.pdf]
